# Supplementary material for: Hot spot prediction in protein-protein interactions by an ensemble system
Source: BMC Syst Biol. 2018 Dec 31;12(Suppl 9):132. doi: 10.1186/s12918-018-0665-8 (PMC6311905; doi:10.1186/s12918-018-0665-8)
Supplement: Supplementary file 2 — All relevant MATLAB code. A simple Matlab implement of our predictor. (ZIP 19,742 kb) [file 12918_2018_665_MOESM2_ESM.zip › Additional file 2/Read ASA/ASA Property/ASEdb/1DX5M.txt]

 NetSurfP - Protein Surface Accessibility and Secondary Structure Predictions

  

|  |  |  |
| --- | --- | --- |
|  |  | NetSurfP - Protein Surface Accessibility and Secondary Structure Predictions  Technical University of Denmark |

  


---

```
# For publication of results, please cite:
# A generic method for assignment of reliability scores applied to solvent accessibility predictions.
# Bent Petersen, Thomas Nordahl Petersen, Pernille Andersen, Morten Nielsen and Claus Lundegaard
# BMC Structural Biology 2009, 9:51 doi:10.1186/1472-6807-9-51
#
# Column 1: Class assignment - B for buried or E for Exposed - Threshold: 25% exposure, but not based on RSA
# Column 2: Amino acid
# Column 3: Sequence name
# Column 4: Amino acid number
# Column 5: Relative Surface Accessibility - RSA
# Column 6: Absolute Surface Accessibility
# Column 7: Z-fit score for RSA prediction
# Column 8: Probability for Alpha-Helix
# Column 9: Probability for Beta-strand
# Column 10: Probability for Coil
E I  1DX5M                  1    0.287  53.095  -2.319   0.005   0.045   0.951
B V  1DX5M                  2    0.156  23.977  -2.326   0.004   0.514   0.481
E E  1DX5M                  3    0.369  64.464  -1.077   0.005   0.336   0.660
B G  1DX5M                  4    0.237  18.620  -1.736   0.020   0.205   0.775
E S  1DX5M                  5    0.559  65.550   1.173   0.021   0.279   0.699
E D  1DX5M                  6    0.579  83.376   1.183   0.020   0.205   0.775
B A  1DX5M                  7    0.054   5.918  -0.054   0.018   0.088   0.893
E E  1DX5M                  8    0.548  95.788   1.468   0.018   0.047   0.935
E I  1DX5M                  9    0.513  94.868   0.097   0.018   0.019   0.964
E G  1DX5M                 10    0.536  42.199  -0.454   0.018   0.019   0.964
E M  1DX5M                 11    0.438  87.664   0.852   0.005   0.015   0.979
B S  1DX5M                 12    0.152  17.826   1.264   0.004   0.138   0.858
B P  1DX5M                 13    0.095  13.410   1.135   0.005   0.336   0.660
B W  1DX5M                 14    0.053  12.867   0.877   0.002   0.816   0.182
B Q  1DX5M                 15    0.050   8.930   0.678   0.006   0.962   0.032
B V  1DX5M                 16    0.019   2.874   1.073   0.001   0.959   0.040
B M  1DX5M                 17    0.029   5.743   0.258   0.001   0.959   0.040
B L  1DX5M                 18    0.017   3.113   1.432   0.001   0.959   0.040
B F  1DX5M                 19    0.093  18.605   0.514   0.001   0.959   0.040
B R  1DX5M                 20    0.152  34.900   0.075   0.001   0.900   0.099
E K  1DX5M                 21    0.378  77.755  -0.051   0.004   0.420   0.576
E S  1DX5M                 22    0.540  63.347  -1.910   0.005   0.045   0.951
E P  1DX5M                 23    0.559  79.393  -1.959   0.005   0.045   0.951
E Q  1DX5M                 24    0.482  86.014  -0.656   0.004   0.085   0.910
E E  1DX5M                 25    0.348  60.726   1.173   0.004   0.197   0.799
B L  1DX5M                 26    0.069  12.689  -0.144   0.004   0.514   0.481
B L  1DX5M                 27    0.084  15.399   0.107   0.003   0.718   0.279
B C  1DX5M                 28    0.014   1.994   1.127   0.021   0.756   0.223
B G  1DX5M                 29    0.039   3.046  -0.791   0.022   0.552   0.426
B A  1DX5M                 30    0.032   3.537  -0.737   0.023   0.655   0.322
B S  1DX5M                 31    0.051   5.930   0.067   0.021   0.756   0.223
B L  1DX5M                 32    0.044   8.130   0.009   0.021   0.756   0.223
B I  1DX5M                 33    0.058  10.767   0.546   0.021   0.451   0.528
B S  1DX5M                 34    0.128  14.955   0.866   0.018   0.088   0.893
E D  1DX5M                 35    0.646  93.103   0.669   0.018   0.047   0.935
E R  1DX5M                 36    0.329  75.295   0.898   0.052   0.084   0.864
B W  1DX5M                 37    0.067  16.138   1.323   0.079   0.592   0.329
B V  1DX5M                 38    0.022   3.320   1.261   0.018   0.846   0.136
B L  1DX5M                 39    0.025   4.504   1.476   0.018   0.846   0.136
B T  1DX5M                 40    0.031   4.369   0.793   0.072   0.855   0.073
B A  1DX5M                 41    0.022   2.435   0.780   0.085   0.775   0.140
B A  1DX5M                 42    0.030   3.273  -0.492   0.079   0.592   0.329
B H  1DX5M                 43    0.061  11.041   0.665   0.074   0.484   0.442
B C  1DX5M                 44    0.015   2.092   1.407   0.074   0.484   0.442
B L  1DX5M                 45    0.048   8.789   0.400   0.066   0.296   0.638
B L  1DX5M                 46    0.264  48.247   0.360   0.056   0.142   0.802
E Y  1DX5M                 47    0.328  70.115  -0.079   0.052   0.084   0.864
E P  1DX5M                 48    0.405  57.484  -0.618   0.053   0.043   0.903
E P  1DX5M                 49    0.514  72.866  -1.483   0.053   0.043   0.903
E W  1DX5M                 50    0.464 111.640  -1.058   0.113   0.043   0.844
E D  1DX5M                 51    0.525  75.725  -1.707   0.053   0.043   0.903
E K  1DX5M                 52    0.520 107.005  -0.995   0.053   0.043   0.903
E N  1DX5M                 53    0.529  77.416  -1.685   0.018   0.088   0.893
E F  1DX5M                 54    0.284  56.919  -0.626   0.018   0.088   0.893
E I  1DX5M                 55    0.393  72.631   0.193   0.005   0.045   0.951
B E  1DX5M                 56    0.166  29.035  -1.257   0.018   0.088   0.893
E N  1DX5M                 57    0.409  59.936  -0.592   0.004   0.197   0.799
E D  1DX5M                 58    0.285  41.097   1.212   0.004   0.514   0.481
B L  1DX5M                 59    0.035   6.409   0.560   0.001   0.900   0.099
B L  1DX5M                 60    0.130  23.748   1.692   0.001   0.959   0.040
B V  1DX5M                 61    0.014   2.213   1.621   0.000   0.983   0.017
B R  1DX5M                 62    0.075  17.083   1.168   0.001   0.959   0.040
B I  1DX5M                 63    0.022   4.051   0.998   0.001   0.959   0.040
B G  1DX5M                 64    0.040   3.148  -1.000   0.002   0.816   0.182
B K  1DX5M                 65    0.162  33.303   0.116   0.023   0.655   0.322
B H  1DX5M                 66    0.146  26.576   0.800   0.021   0.756   0.223
E S  1DX5M                 67    0.292  34.211   0.021   0.022   0.552   0.426
B R  1DX5M                 68    0.127  28.969  -0.030   0.022   0.359   0.619
E T  1DX5M                 69    0.393  54.454  -0.587   0.021   0.279   0.699
E R  1DX5M                 70    0.476 109.004   0.413   0.020   0.205   0.775
E Y  1DX5M                 71    0.493 105.290  -0.892   0.018   0.088   0.893
E E  1DX5M                 72    0.575 100.487  -1.383   0.018   0.047   0.935
E R  1DX5M                 73    0.744 170.399  -1.116   0.018   0.047   0.935
E N  1DX5M                 74    0.468  68.515  -1.146   0.018   0.047   0.935
E I  1DX5M                 75    0.372  68.839  -0.431   0.004   0.197   0.799
E E  1DX5M                 76    0.403  70.491  -0.774   0.004   0.616   0.381
B K  1DX5M                 77    0.207  42.621   0.728   0.001   0.900   0.099
E I  1DX5M                 78    0.422  78.033   0.455   0.001   0.959   0.040
B S  1DX5M                 79    0.105  12.259   1.660   0.001   0.959   0.040
E M  1DX5M                 80    0.398  79.680   1.495   0.001   0.900   0.099
B L  1DX5M                 81    0.024   4.358   0.609   0.002   0.816   0.182
E E  1DX5M                 82    0.431  75.296   1.265   0.001   0.959   0.040
E K  1DX5M                 83    0.318  65.330   1.336   0.001   0.959   0.040
B I  1DX5M                 84    0.061  11.230   0.734   0.001   0.959   0.040
B Y  1DX5M                 85    0.122  26.114   1.112   0.001   0.959   0.040
B I  1DX5M                 86    0.195  36.038   0.670   0.002   0.816   0.182
B H  1DX5M                 87    0.060  10.878   0.749   0.005   0.262   0.733
E P  1DX5M                 88    0.441  62.535   0.418   0.005   0.045   0.951
E R  1DX5M                 89    0.521 119.378   0.096   0.005   0.045   0.951
B Y  1DX5M                 90    0.091  19.340   0.061   0.005   0.045   0.951
E N  1DX5M                 91    0.367  53.714   0.291   0.018   0.047   0.935
E W  1DX5M                 92    0.505 121.452  -0.546   0.018   0.019   0.964
E R  1DX5M                 93    0.652 149.216  -1.309   0.018   0.019   0.964
E E  1DX5M                 94    0.584 102.042  -0.670   0.018   0.019   0.964
E N  1DX5M                 95    0.347  50.845  -0.471   0.018   0.019   0.964
B L  1DX5M                 96    0.171  31.347   0.504   0.018   0.047   0.935
E D  1DX5M                 97    0.435  62.669  -0.212   0.018   0.047   0.935
B R  1DX5M                 98    0.073  16.763   0.443   0.018   0.088   0.893
B D  1DX5M                 99    0.036   5.188  -0.324   0.021   0.451   0.528
B I  1DX5M                100    0.024   4.422   1.202   0.018   0.846   0.136
B A  1DX5M                101    0.017   1.895   1.213   0.011   0.918   0.071
B L  1DX5M                102    0.019   3.534   1.459   0.011   0.918   0.071
B M  1DX5M                103    0.027   5.423   1.063   0.011   0.918   0.071
B K  1DX5M                104    0.201  41.387   1.682   0.001   0.900   0.099
B L  1DX5M                105    0.033   6.061  -0.006   0.003   0.718   0.279
E K  1DX5M                106    0.493 101.328   1.202   0.021   0.279   0.699
E K  1DX5M                107    0.526 108.136   0.753   0.020   0.205   0.775
E P  1DX5M                108    0.399  56.561   0.254   0.020   0.205   0.775
B V  1DX5M                109    0.063   9.637   0.059   0.021   0.279   0.699
E A  1DX5M                110    0.584  64.324   1.437   0.022   0.359   0.619
B F  1DX5M                111    0.264  53.025   0.051   0.020   0.205   0.775
E S  1DX5M                112    0.315  36.918  -0.548   0.018   0.047   0.935
E D  1DX5M                113    0.657  94.703  -0.290   0.018   0.047   0.935
E Y  1DX5M                114    0.306  65.349   0.575   0.005   0.262   0.733
B I  1DX5M                115    0.044   8.048   0.341   0.003   0.718   0.279
B H  1DX5M                116    0.207  37.635   1.051   0.002   0.816   0.182
B P  1DX5M                117    0.068   9.607  -0.082   0.018   0.846   0.136
B V  1DX5M                118    0.018   2.720   1.402   0.018   0.846   0.136
B C  1DX5M                119    0.053   7.483  -0.312   0.003   0.718   0.279
B L  1DX5M                120    0.030   5.511   0.688   0.005   0.336   0.660
B P  1DX5M                121    0.087  12.317   0.130   0.005   0.015   0.979
E D  1DX5M                122    0.344  49.614   0.725   0.018   0.019   0.964
E R  1DX5M                123    0.496 113.515  -0.341   0.058   0.017   0.925
E E  1DX5M                124    0.634 110.812  -2.049   0.115   0.016   0.868
E T  1DX5M                125    0.361  50.057  -1.024   0.113   0.043   0.844
E A  1DX5M                126    0.481  53.006  -1.706   0.113   0.043   0.844
E A  1DX5M                127    0.477  52.587  -0.928   0.053   0.043   0.903
E S  1DX5M                128    0.580  67.976  -0.482   0.053   0.043   0.903
E L  1DX5M                129    0.457  83.603   0.148   0.052   0.084   0.864
B L  1DX5M                130    0.072  13.220  -0.701   0.018   0.047   0.935
E Q  1DX5M                131    0.423  75.494  -0.050   0.018   0.019   0.964
E A  1DX5M                132    0.561  61.844  -0.544   0.018   0.019   0.964
E G  1DX5M                133    0.518  40.774  -1.084   0.005   0.015   0.979
B Y  1DX5M                134    0.159  33.957  -0.084   0.005   0.045   0.951
E K  1DX5M                135    0.326  67.058   1.746   0.004   0.616   0.381
B G  1DX5M                136    0.015   1.188   1.425   0.001   0.959   0.040
B R  1DX5M                137    0.085  19.534   1.047   0.001   0.959   0.040
B V  1DX5M                138    0.015   2.259   1.196   0.001   0.959   0.040
B T  1DX5M                139    0.039   5.451   0.402   0.001   0.900   0.099
B G  1DX5M                140    0.030   2.322  -1.325   0.002   0.816   0.182
B W  1DX5M                141    0.086  20.755   0.133   0.021   0.451   0.528
B G  1DX5M                142    0.041   3.203  -1.779   0.022   0.359   0.619
E N  1DX5M                143    0.264  38.591   0.561   0.022   0.359   0.619
B L  1DX5M                144    0.111  20.361   0.330   0.021   0.279   0.699
E K  1DX5M                145    0.393  80.737   0.276   0.064   0.216   0.721
E E  1DX5M                146    0.535  93.447  -0.990   0.019   0.141   0.840
E T  1DX5M                147    0.511  70.931  -1.707   0.018   0.088   0.893
E W  1DX5M                148    0.473 113.732  -1.535   0.018   0.088   0.893
E T  1DX5M                149    0.506  70.154  -1.979   0.019   0.141   0.840
E A  1DX5M                150    0.474  52.235  -1.731   0.056   0.142   0.802
E N  1DX5M                151    0.432  63.172  -1.595   0.052   0.084   0.864
E V  1DX5M                152    0.490  75.298  -1.816   0.053   0.043   0.903
E G  1DX5M                153    0.596  46.882  -2.693   0.018   0.019   0.964
E K  1DX5M                154    0.340  70.000  -1.814   0.018   0.019   0.964
E G  1DX5M                155    0.538  42.348  -1.346   0.018   0.019   0.964
E Q  1DX5M                156    0.293  52.401  -0.378   0.058   0.017   0.925
B P  1DX5M                157    0.096  13.651  -0.613   0.115   0.016   0.868
E S  1DX5M                158    0.487  57.076  -0.110   0.268   0.043   0.689
B V  1DX5M                159    0.222  34.198   1.422   0.199   0.152   0.649
B L  1DX5M                160    0.053   9.778   0.452   0.135   0.317   0.548
B Q  1DX5M                161    0.241  43.078   0.665   0.079   0.592   0.329
E V  1DX5M                162    0.332  51.044   0.925   0.011   0.918   0.071
B V  1DX5M                163    0.027   4.211   0.563   0.011   0.918   0.071
E N  1DX5M                164    0.477  69.862   0.594   0.018   0.846   0.136
B L  1DX5M                165    0.041   7.434   0.760   0.023   0.655   0.322
E P  1DX5M                166    0.350  49.665   0.704   0.023   0.655   0.322
B I  1DX5M                167    0.172  31.765  -0.146   0.004   0.616   0.381
B V  1DX5M                168    0.168  25.837   0.972   0.004   0.514   0.481
E E  1DX5M                169    0.448  78.301   1.041   0.004   0.197   0.799
E R  1DX5M                170    0.274  62.631   0.794   0.694   0.003   0.303
E P  1DX5M                171    0.725 102.835   1.387   0.858   0.002   0.139
E V  1DX5M                172    0.403  62.018   2.269   0.858   0.002   0.139
B C  1DX5M                173    0.015   2.036   1.113   0.879   0.010   0.111
E K  1DX5M                174    0.364  74.916   1.218   0.879   0.010   0.111
E D  1DX5M                175    0.675  97.224   1.119   0.802   0.014   0.185
E S  1DX5M                176    0.304  35.676   1.053   0.782   0.003   0.216
B T  1DX5M                177    0.134  18.516   0.151   0.600   0.003   0.397
E R  1DX5M                178    0.627 143.491   0.069   0.246   0.004   0.750
E I  1DX5M                179    0.547 101.214  -1.199   0.115   0.016   0.868
E R  1DX5M                180    0.485 111.157   1.189   0.113   0.043   0.844
B I  1DX5M                181    0.043   7.955  -0.349   0.053   0.043   0.903
E T  1DX5M                182    0.289  40.112   1.076   0.018   0.019   0.964
E D  1DX5M                183    0.590  85.019   0.261   0.018   0.019   0.964
E N  1DX5M                184    0.301  44.096   0.135   0.018   0.019   0.964
B M  1DX5M                185    0.026   5.303   0.612   0.021   0.279   0.699
B F  1DX5M                186    0.030   5.961   0.579   0.021   0.756   0.223
B C  1DX5M                187    0.013   1.797   1.328   0.021   0.756   0.223
B A  1DX5M                188    0.022   2.369   0.577   0.022   0.552   0.426
B G  1DX5M                189    0.049   3.880  -0.889   0.022   0.359   0.619
B Y  1DX5M                190    0.178  38.060   0.468   0.021   0.279   0.699
B K  1DX5M                191    0.279  57.349   0.299   0.020   0.205   0.775
E P  1DX5M                192    0.562  79.805  -0.768   0.019   0.141   0.840
E D  1DX5M                193    0.590  85.048  -1.407   0.018   0.047   0.935
E E  1DX5M                194    0.632 110.358  -2.270   0.018   0.019   0.964
E G  1DX5M                195    0.570  44.867  -1.204   0.018   0.019   0.964
E K  1DX5M                196    0.444  91.351  -1.188   0.018   0.019   0.964
E R  1DX5M                197    0.359  82.257  -1.124   0.018   0.047   0.935
E G  1DX5M                198    0.338  26.585   0.314   0.019   0.141   0.840
B D  1DX5M                199    0.065   9.424  -2.373   0.020   0.205   0.775
B A  1DX5M                200    0.071   7.780  -0.418   0.021   0.279   0.699
B C  1DX5M                201    0.026   3.608  -0.338   0.021   0.279   0.699
E E  1DX5M                202    0.288  50.349   0.462   0.020   0.205   0.775
B G  1DX5M                203    0.154  12.120  -1.264   0.019   0.141   0.840
B D  1DX5M                204    0.172  24.857  -0.103   0.018   0.047   0.935
B S  1DX5M                205    0.183  21.424  -1.938   0.018   0.019   0.964
B G  1DX5M                206    0.169  13.269  -2.768   0.018   0.019   0.964
B G  1DX5M                207    0.089   6.989  -0.446   0.018   0.088   0.893
B P  1DX5M                208    0.086  12.203  -0.899   0.021   0.279   0.699
B F  1DX5M                209    0.054  10.878   0.027   0.004   0.616   0.381
B V  1DX5M                210    0.056   8.638   0.450   0.021   0.756   0.223
B M  1DX5M                211    0.039   7.884   0.107   0.021   0.756   0.223
E K  1DX5M                212    0.358  73.599   0.339   0.004   0.616   0.381
B S  1DX5M                213    0.279  32.699  -0.798   0.004   0.420   0.576
E P  1DX5M                214    0.519  73.674  -0.580   0.004   0.197   0.799
E F  1DX5M                215    0.547 109.863  -1.500   0.004   0.085   0.910
E N  1DX5M                216    0.681  99.640  -1.095   0.005   0.015   0.979
E N  1DX5M                217    0.432  63.186  -1.091   0.005   0.045   0.951
E R  1DX5M                218    0.287  65.838   1.743   0.004   0.514   0.481
B W  1DX5M                219    0.072  17.364   0.748   0.001   0.900   0.099
B Y  1DX5M                220    0.094  20.173   1.545   0.001   0.959   0.040
B Q  1DX5M                221    0.024   4.233   0.600   0.001   0.959   0.040
B M  1DX5M                222    0.045   8.924   1.059   0.001   0.959   0.040
B G  1DX5M                223    0.030   2.353  -0.272   0.011   0.918   0.071
B I  1DX5M                224    0.032   5.883   0.511   0.011   0.918   0.071
B V  1DX5M                225    0.036   5.579   0.354   0.021   0.756   0.223
B S  1DX5M                226    0.039   4.524  -0.621   0.021   0.756   0.223
B W  1DX5M                227    0.130  31.361   0.233   0.021   0.451   0.528
B G  1DX5M                228    0.093   7.351  -0.889   0.064   0.216   0.721
E E  1DX5M                229    0.366  64.010   0.418   0.056   0.142   0.802
E G  1DX5M                230    0.321  25.239  -1.202   0.052   0.084   0.864
B C  1DX5M                231    0.068   9.533  -0.342   0.053   0.043   0.903
B D  1DX5M                232    0.220  31.702  -1.039   0.058   0.017   0.925
E R  1DX5M                233    0.478 109.439   0.654   0.018   0.019   0.964
E D  1DX5M                234    0.580  83.564   0.237   0.018   0.019   0.964
E G  1DX5M                235    0.640  50.392  -0.991   0.003   0.003   0.994
E K  1DX5M                236    0.297  61.093   1.067   0.018   0.019   0.964
B Y  1DX5M                237    0.117  25.046  -0.695   0.018   0.047   0.935
B G  1DX5M                238    0.046   3.652  -2.338   0.022   0.359   0.619
B F  1DX5M                239    0.038   7.707   0.025   0.021   0.756   0.223
B Y  1DX5M                240    0.090  19.169   0.064   0.085   0.775   0.140
B T  1DX5M                241    0.037   5.187  -0.149   0.087   0.683   0.230
B H  1DX5M                242    0.208  37.890   0.560   0.159   0.516   0.325
B V  1DX5M                243    0.036   5.456   0.426   0.428   0.171   0.402
B F  1DX5M                244    0.269  53.928   0.360   0.660   0.049   0.291
E R  1DX5M                245    0.344  78.684   0.869   0.622   0.015   0.363
B L  1DX5M                246    0.059  10.840   0.254   0.802   0.014   0.185
B K  1DX5M                247    0.192  39.536   1.213   0.938   0.007   0.055
E K  1DX5M                248    0.569 117.002   1.513   0.970   0.001   0.030
B W  1DX5M                249    0.113  27.104   0.965   0.970   0.001   0.030
B I  1DX5M                250    0.021   3.811   0.948   0.988   0.000   0.012
E Q  1DX5M                251    0.383  68.404   1.825   0.988   0.000   0.012
E K  1DX5M                252    0.532 109.474   1.938   0.988   0.000   0.012
B V  1DX5M                253    0.181  27.850   1.626   0.970   0.001   0.030
B I  1DX5M                254    0.059  10.970   0.439   0.923   0.002   0.076
E D  1DX5M                255    0.594  85.595   1.486   0.923   0.002   0.076
E Q  1DX5M                256    0.665 118.805   1.163   0.782   0.003   0.216
E F  1DX5M                257    0.453  90.937  -0.927   0.502   0.002   0.495
E G  1DX5M                258    0.456  35.864  -2.085   0.109   0.005   0.886
E E  1DX5M                259    0.876 153.037   1.367   0.003   0.003   0.994

Explain the output.  Go back.
```

---
